# Integration of diverse DNA substrates by a casposase can be targeted to R-loops *in vitro* by its fusion to Cas9

**DOI:** 10.1042/BSR20203595

**Published:** 2021-01-05

**Authors:** Chun Hang Lau, Edward L. Bolt

**Affiliations:** School of Life Sciences, University of Nottingham, Nottingham NG7 2UH, U.K.

**Keywords:** casposase, CRISPR, DNA integration

## Abstract

CRISPR systems build adaptive immunity against mobile genetic elements by DNA capture and integration catalysed by Cas1–Cas2 protein complexes. Recent studies suggested that CRISPR repeats and adaptation module originated from a novel type of DNA transposons called casposons. Casposons encode a Cas1 homologue called casposase that alone integrates into target molecules single and double-stranded DNA containing terminal inverted repeats (TIRs) from casposons. A recent study showed *Methanosarcina mazei* casposase is able to integrate random DNA oligonucleotides, followed up in this work using *Acidoprofundum boonei* casposase, from which we also observe promiscuous substrate integration. Here we first show that the substrate flexibility of *Acidoprofundum boonei* casposase extends to random integration of DNA without TIRs, including integration of a functional gene. We then used this to investigate targeting of the casposase-catalysed DNA integration reactions to specific DNA sites that would allow insertion of defined DNA payloads. Casposase–Cas9 fusions were engineered that were catalytically proficient *in vitro* and generated RNA-guided DNA integration products from short synthetic DNA or a gene, with or without TIRs. However, DNA integration could still occur unguided due to the competing background activity of the casposase moiety. Expression of Casposase-dCas9 in *Escherichia coli* cells effectively targeted chromosomal and plasmid *lacZ* revealed by reduced β-galactosidase activity but DNA integration was not detected. The promiscuous substrate integration properties of casposases make them potential DNA insertion tools. The Casposase–dCas9 fusion protein may serves as a prototype for development in genetic editing for DNA insertion that is independent of homology-directed DNA repair.

## Introduction

Horizontal gene transfer between prokaryotic cells is a major driving force for their evolution but is also resisted by host self-defence proteins that have evolved to maintain the genetic *status quo* [1–3]. CRISPR–Cas systems provide adaptive immune defence in bacteria and archaea [4–6]. CRISPR–Cas systems centre on specialised chromosomal sites comprising a CRISPR DNA array and *cas* (CRISPR-associated) genes. The CRISPR locus is a depot of 25–40 base pair DNA ‘spacer’ fragments acquired in cells during previous encounters with DNA from mobile genetic elements (MGEs). The spacer sequences in CRISPRs alternate with DNA ‘repeats’ that provide sequence and structural elements necessary for correct functioning of the CRISPR locus. Transcription of CRISPRs, and processing of the RNA transcripts, generates crRNAs that each contain one spacer sequence. A crRNA can be targeted to MGE DNA by base pairing catalysed by Cas ‘Interference’ protein complexes, which include nucleases to destroy MGE DNA, thus mounting an immunity-based defence.

Acquisition of new DNA spacers into a CRISPR locus therefore underpins immunity by providing substrates for effective interference. Cas1–Cas2 proteins, aided by host non-Cas proteins, capture MGE DNA ‘pre-spacer’ or ‘protospacer’ fragments, and integrate them into CRISPRs, processes called ‘Adaptation’ [7,8]. The DNA integration reaction into CRISPR centres on Cas1-catalysed transesterfication for joining pre-spacer DNA with CRISPR DNA [9–11], resulting in DNA gaps that are filled by DNA polymerase, duplicating the DNA repeat [12].

Cas1 homologues were identified within transposons called ‘casposons’ [13]. These are proposed to be antecedent of CRISPR–cas systems – the Cas1 homologues, ‘casposases’, catalyse DNA transposition reactions that are thought to have evolved into CRISPR adaptation catalysed by Cas1–Cas2 complexes [14,15]. The first analysis of a casposase *in vitro*, from the archaeon *Aciduliprofundum boonei*, identified single-end and double-end integration of short (15–30 base pair) duplex DNA, and of a kanamycin resistance gene as part of an artificial mini-casposon [16]. The integrated DNA was constructed to contain terminal inverted repeat (TIR) DNA sequences, which are present at casposon ends within the genome of *A. boonei*. Mini-casposon integration resulted in 14–15 base pair target site duplications (TSDs) of DNA [16]. Subsequent analyses of *A. boonei* casposase, and of casposases from the archaeal organisms *Methanosarcina mazei* and *Nitrosopumilus koreensis*, showed integration of diverse nucleic acid substrates, including single-stranded DNA and RNA, if an integration target site was included in reactions [15,17,18]. The target sites vary according to the casposase; DNA sequence close to the tRNA-Pro gene for *A. boonei* [17], the tRNA-Leu gene in the archaeon *M. mazei* [15] or the gene encoding protein EF2 in *N. koreensis*. Although casposases integrate DNA into a plasmid (pUC19) at random sites, integration is improved at the appropriate target sequence.

The broad-range of DNA substrates integrated by casposases is potentially useful for genetic editing if it can be directed to defined sites by an R-loop formed by an effector such as Cas9. In many genetic editing procedures Cas9 forms a site-specific DNA double-strand break at the R-loop, initiating insertion of new DNA sequence dependent on host cell enzymes of homologous recombination (HR) [19–21]. The effectiveness of HR for genetic editing is offset by drawbacks including the triggering of unpredictable genome re-arrangements and low efficacy. Other strategies have sought to overcome the problems of HR-dependent genetic insertion by exploiting interactions or fusions of Cas9 with transposases [22–25], and by re-purposing group-II introns [26]. We were interested in casposases for this purpose because of their ability to integrate a wide variety of DNA substrates. We present data that casposase from *A. boonei* integrates DNA without TIRs. By bioengineering a fusion of *A. boonei* casposase with bacterial Cas9 we could control the DNA integration activity *in vitro* by targeting it to sites dictated by sgRNAs. Expression of Casp–Cas9 in *E. coli* cells was effective for inactivating *lacZ*. Casp–Cas9 therefore has potential for RNA-guided genetic editing that can insert DNA sequences independently of the usual need for homology-directed DNA processes.

## Materials and methods

### Molecular cloning

Details of DNA plasmids, primers and oligonucleotides used to form sgRNA are in Supplementary Tables S1–3 and Figure S1. *Aciduliprofundum boonei* T469 genomic DNA purchased from DSMZ was used as a template for PCR amplification of its casposase gene (NCBI gene ID 8827333, annotated as ‘Cas1’ in this organism NCBI) which was cloned into pACYC-Duet using BamHI and PstI restriction sites to generate new plasmid pCL2. The gene encoding *Streptococcus pyogenes* Cas9 was amplified from pCas9 (Addgene) for cloning into pCL2 downstream of the *casposase* gene, between PstI and NotI restriction sites of the plasmid. The sequence between the last codon of casposase, removing the stop codon, and Cas9 was replaced by flexible linkers of 24-residue (GGS)_8_ using Q5 site directed mutagenesis (New England Biolabs, NEB). The same strategy was used to construct the Casp–dCas9 fusion protein from pdCas9 plasmid (AddGene). Plasmids pAB and pABmut were synthesised by GeneArt™ (Life Technologies) and the plasmid backbond was pMA-T. pAB contains 250 bp sequence from pACYC-Duet that flanks the sgRNA-binding site, and pABmu contains the same sequence except having the integration site A and site B mutated.

For experiments targeting dCas9 or Casp–dCas9 to *Escherichia coli lacZ*, we used the Q5 site-directed mutagenesis procedures (New England Biolabs) to insert necessary DNA sequence for the sgRNA-*lacZ* into pgRNA-bacteria (Addgene). The DNA encoding sgRNA-*lacZ* RNA was then PCR amplified and subcloned into pCDF-1b between PstI and XbaI sites to generate a sgRNA-*lacZ* expressing plasmid that is compatible with protein expressing plasmids for antibiotic selection and origin of replication. For plasmids expressing protein and sgRNA-*lacZ* (pCL5 and pCL6), the DNA encoding sgRNA-*lacZ* and its promoter was subcloned into fusion protein expressing plasmid.

### Purification of Casposase, Cas9, Casp–Cas9 and SSB proteins

Purified proteins are shown in Supplementary Figure S2. Hexa-histidine tagged ((His)_6_) casposase, Cas9, dCas9 and the fusion proteins Casp-Cas9/dCas9 were expressed and purified using the same method. Proteins were expressed in BL21 AI *E. coli* grown at 37°C until optical density (OD_600nm_) had reached 0.6, for addition of arabinose (0.1% w/v) and IPTG (0.5 mM) to induce protein expression overnight at 18°C. Cells were pelleted and resuspended in buffer A, containing 25 mM Tris pH7.5, 500 mM NaCl, 20 mM imidazole and an EDTA-free protease inhibitor cocktail (Roche). Resuspended cells were lysed by sonication, and soluble proteins were obtained by centrifugation at 35,000 ***g*** for 30 min at 4°C. Soluble proteins were loaded onto a Ni^2+^ charged HP-column (GE Healthcare) that was washed with buffer A and the (His)_6_ proteins were eluted by a linear gradient of increasing imidazole (0–500 mM) in buffer A. Fractions containing the desired protein, as determined by SDS-PAGE and coomassie staining, were dialysed at 4°C against buffer HA (25 mM Tris pH7.5, 150 mM NaCl, 10% glycerol) then loaded onto a 5 ml HiTrap heparin column (GE Healthcare), washed by buffer HA and eluted by a gradient of 0.15–1 M NaCl in buffer HA. Fractions containing desired protein were concentrated by centrifugal filter (Millipore) and buffer was exchanged for storage in 25 mM Tris pH7.5, 500 mM NaCl, 15% glycerol. *Escherichia coli* SSB protein was a kind gift from Prof. Peter McGlynn (University of York), and had been purified according to the protocol in [27].

### DNA disintegration and integration assays

DNA oligonucleotides were purchased from Sigma-Aldrich (Supplementary Table S3), and structures of DNA substrates are illustrated in Supplementary Figure S1. DNA was imaged by use of either ethidium bromide DNA staining within agarose gels, or by Cy5 end labels to DNA strands. Small synthetic DNA substrates were formed by annealing a 5′-Cy5 labelled oligonucleotide with unlabelled oligonucleotide in annealing buffer (10 mM Tris pH7.5, 50 mM NaCl, 1 mM EDTA). The substrate was isolated from unannealed DNA strand by PAGE in a 10% acrylamide TBE gel, visualising the fully formed substrate by the presence of Cy5 and excising the corresponding gel slice for elution into 20 mM Tris pH7.5 and 150 mM NaCl.

Disintegration assays used 25 nM of DNA fork-3 and 150 nM of protein in buffer I (25 mM Tris pH 7.5, 106 mM KCl, 50 μg/ml BSA, 5 mM MnCl_2_, 2 mM DTT, 9% glycerol) incubating for 1 h at 37°C. Reactions were stopped by adding proteinase K buffer (10 mM Tris pH8, 24 U proteinase K (Invitrogen), 20 mM EDTA) and incubating at 37°C for 30 min. Formamide loading dye (80% formamide and 20% glycerol supplemented with dye Orange G) was added to reactions and the samples were heated at 95°C for 10 min for running in 20% acrylamide 7 M urea TBE gels in TBE buffer at 7W for 2 h. Gels were analysed directly using a Typhoon Biomolecular Imager (GE Healthcare).

**Figure 1:**
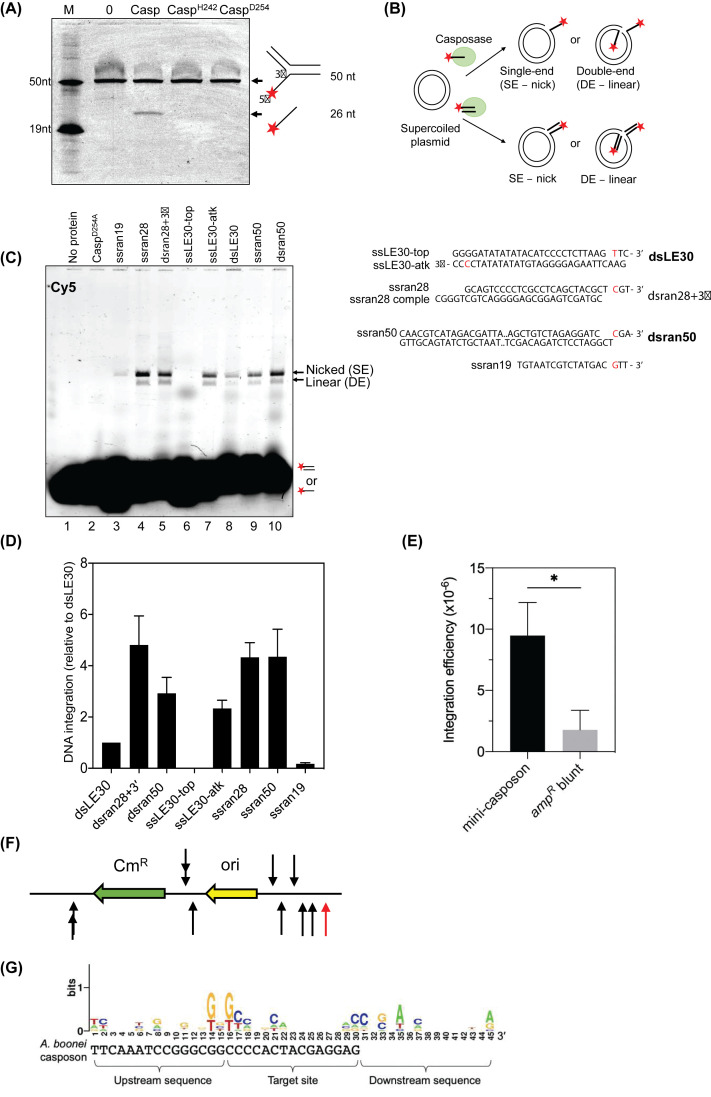
*A. boonei* casposase catalyses integration of ssDNA and dsDNA with and without TIRs (**A**) Denaturing acrylamide gel showing casposase-catalysed disintegration of a DNA flap from fork-3 DNA. Reactions lasted 1 h and contained 25 nM of fork-3 DNA and 150 nM of casposase wild-type or mutant proteins as indicated. (**B**) The cartoon illustrates the integration assay and its outcomes. The agarose gel is a representative summary of Cy5-labelled single-end (nicked) and double-end (linear) integration products formed from integration into pACYC-Duet of substrates (100 nM) indicated above each lane, by 150 nM of casposase or inactive casposase mutant D254A for 1 h. (**C**) Measurement of relative integration activities of casposase (150 nM) on ssDNA or dsDNA substrates (100 nM) into pACYC-Duet (150 ng). Reactions were in triplicate to quantify integration relative to the dsLE30 substrate, showing distribution of standard error from the calculated mean. The DNA integration substrates are shown labelled and the box highlights the presence of the significant cytosine at the -3 position from the DNA 3′ end. (**D**) Average integration efficiency of DNA-*bla* with or without TIRs into pACYC-Duet catalysed by casposase. Results were from three independent repeats and full data were shown in Supplementary Figure S6. Error bars standard error of the mean. Unpaired *t* test showed the difference between mini-casposon integration and DNA-*bla* blunt was significant (*P*=0.0131); *: *P*-value<0.05. (**E**) Location of integration sites of mini-casposon (black arrows) and DNA-*bla* without TIRs (red arrow) in pACYC-Duet. Ten mini-casposon integrated pACYC-Duet plasmids were sequenced and one for DNA-*bla*. Direction of arrows indicates the direction of insertion of the 3′-OH of the left end nucleophilic attack strand into the plus strand or the minus strand. (**F**) DNA logo plot of pre-integration site generated from 10 sequenced mini-casposon integration sites in pACYC-Duet. Nucleotides 1–15 were upstream sequence and nucleotides 16–30 were target site which would be duplicated after integration. Nucleotides 31–45 were downstream sequence. The *A. boonei* T469 casposon insertion site (NC 013926.1) was shown below the DNA logo for comparison.

Integration assays without sgRNA used 100 nM Cy5-labelled DNA substrate, 150 ng of pACYC-Duet plasmid DNA and protein at 150 nM, unless stated in results, in buffer I for 1 h at 37°C. Integration assays that included sgRNA were carried out step-wise, first incubating the protein with 300 nM sgRNA at 37°C for 10 min, then addition of 150 ng of target plasmid for 30 min. To this mixture was added the Cy5-labelled DNA for 1 h. Reactions were stopped in the same way as for disintegration assays and run in 0.7% agarose gels in 1× TBE at 120V for 70 min. Gels were imaged first by the Typhoon Imager to observe integration of fluorescent substrate into the plasmid. Then gels were stained with ethidium bromide to observe plasmid DNA using a UV-transilluminator (Syngene). Relative efficiencies of Cy5-DNA integration (e.g. in Figure 1C) were measured using ImageQuant software on the JPEG file of the gel image, using the no protein lane as a baseline for zero integration. To assess integration products by PCR, gel bands corresponding to Cy5-incorporated plasmid DNA were excised and DNA was extracted using a Qiagen kit. PCR was used to amplify the integrated DNA region, and PCR products were analysed in 0.8% agarose gels by ethidium bromide staining. DNA corresponding to targeted integration was excised, purified and Sanger sequenced.

For integration reactions using larger DNA substrates based on the *A. boonei* mini-casposon, the DNA was constructed by inserting 38 bp terminal inverted repeat (TIR) sequences derived from the *A. boonei* casposon into pET14b plasmid either side of the ampicillin resistance gene using Q5 site-directed mutagenesis. The resulting 1.2 kb DNA was amplified by PCR and gel purified for use in integration reactions as described above. The ampicillin resistance gene DNA lacking TIRs was created by amplification of the gene *bla* from pET14b and used directly in assays after purification from agarose gels. Reactions contained 45 ng of mini-casposon, DNA-*bla* and 150 ng pACYC-Duet plasmid DNA and were incubated at 37°C overnight. Reactions were stopped as before, products ethanol precipitated and resuspended in dH_2_O for use in PCR templates or transformation to detect integration.

### R-loop and DNA-binding assays

R-loop formation was assessed against dsran50 DNA using a targeting sgran50. This sgRNA was generated by PCR creating a DNA duplex that included the T7 promoter sequence, the DNA encoding the sgran50 and the DNA sequence forming the sgRNA scaffold. The DNA was transcribed using HiScribe™ T7 High Yield RNA Synthesis Kit (NEB) and treated with RNase-free RQ1 DNase (Promega). sgRNA was run on 8% acrylamide 7 M urea TBE gel, visualised by UV shadowing and excised for elution in RNase-free water at 4°C for 24 h. The sgRNA was concentrated by ethanol precipitation and its concentration determined by absorbance at 260 nm. The R-loop forming assays contained 150 nM proteins incubated with 300 nM sgRNA (at 37°C for 10 min in binding buffer (25 mM Tris pH 7.5, 106 mM KCl, 50 μg/ml BSA, 5 mM EDTA, 2 mM DTT, 9% glycerol). Then 25 nM Cy5 labelled target dsran50 and 75 nM unlabelled competitor MW12 dsDNA were added to the sgRNA-Cas9 complex and incubated at 37°C for 20 min. The reaction was stopped by adding protease K stop buffer and was run through 8% acrylamide TBE gels followed by imaging using a Typhoon scanner. DNA/R-loop binding reactions were done in the same way but without adding proteinase K stop buffer. R-loop formation against *lacZ* of 150 ng pf pUC19 was assessed in the same way but electrophoresis was in 0.8% TAE agarose gels at 10 V overnight.

### Miller assays measuring β-galactosidase activity

For *in vivo* targeting of *lacZ* on a plasmid, *E. coli* strain CL003 (Supplementary Table S4) was transformed with pRC7 (Supplementary Table S1), which contains the *lac* operon and confers ampicillin resistance, and plasmid encoding sgRNA-*lacZ* and a fusion protein (Supplementary Table S3). For targeting of *lacZ* on a chromosome, *E. coli* strain EB377 was transformed with plasmid encoding a sgRNA-*lacZ* and a plasmid for protein expression. A single colony was inoculated into 5 ml LB containing antibiotics to establish an overnight culture, from which 50 μl was inoculated into 5 ml of fresh LB also containing antibiotics for growth at 37°C until OD_600nm_ of 0.2. L-arabinose (0.1%) and IPTG (0.5 mM) were added to induce protein and sgRNA expression and growth was continued until OD_600nm_ reached 0.6. The Miller assay [28] was used in modified form: Cells were pelleted and resuspended in the same volume of cold Z buffer (60 mM Na_2_HPO_4_, 40 mM NaH_2_PO_4_, 10 mM KCl, 1 mM MgSO_4_, 50 mM β-mercaptoethanol). The OD_600nm_ of cells in Z buffer was recorded. Resuspended cells (0.5 ml) were mixed with 0.5 ml Z buffer and these 2-fold diluted cells were permeabilised with 100 µl chloroform and 50 µl 0.1% SDS. To this was added 200 µl of 4 mg/ml ONPG in phosphate buffer (60 mM Na_2_HPO_4_, 40 mM NaH_2_PO_4_, pH 7.0) and incubated at 28°C for 12 min. The reaction was stopped by adding 0.5 ml of 1 M Na_2_CO_3_. The reaction was centrifuged to remove debris and chloroform and OD_420nm_ and OD_550nm_ of the supernatant was recorded. The Miller unit was calculated as: Miller units = 1000 × (OD_420_ - 1.75 × OD_550_)/(T × V × OD_600_), where *T* was the reaction time in minutes and *V* is the volume of culture used in the assay, in millilitres.

Casp–dCas9 catalysed DNA integration *in vivo* was attempted by growing and inducing cells as described for the Miller assay, before making cells electrocompetent at OD_600_ = 0.6. Cells were electroporated with dsran28+3′ (3 μM) or DNA-*bla* (150 ng) and recovered for 1 h in SOC medium. Cells were spread onto LB or LB plates containing ampicillin after electroporation with dsran28+3′ or DNA-*bla*, respectively. Cells were diluted 100-fold for PCR to detect integration.

### Preparation of human cell-free extract

Human osteosarcoma U2OS cells were cultured in DMEM complete medium and 1.6 × 10^7^ cells were harvested. Cells were washed in cold hypotonic buffer (20 mM HEPES pH7.5, 5 mM KCl, 1.5 mM MgCl2, 1 mM DTT) containing 1× protease inhibitor cocktail. Cells were pelleted again and resuspended in cold hypotonic buffer, followed by incubation on ice for 15 min. A 1 ml syringe and a 25G needle were used to homogenise cells with 20 strokes. The cell lysate was left on ice for 60 min to allow release of nuclear proteins and then centrifuged at 12,000 ***g*** for 15 min. The supernatant containing proteins was aliquoted and stored at −80°C. The concentration of cell-free extracts was determined using a Bradford assay.

## Results

### *A. boonei* casposase disintegrates and integrates DNA substrates without terminal inverted repeat sequences

The casposase homologue Cas1 is a DNA integrase when complexed with Cas2 in CRISPR systems, and catalyses DNA disintegration reactions without Cas2 [9,29]. Disintegration of branched DNA molecules is observed as DNA strand breakage after nucleophilic attack from a proximal 3′-OH, a reaction summarised in Figure 1A. Purified *A. boonei* casposase (Supplementary Figure S2A) catalysed disintegration of a DNA fork (Fork-3) more effectively in manganese compared with magnesium (Figure 1A and Supplementary Figure S3, consistent with DNA integration by casposase and *Pseudomonas aeruginosa* Cas1 [16,30]. Casposase active site mutants H242A and D254A were catalytically inactive (Figure 1A). In these disintegration assays the DNA fork substrate lacks terminal inverted repeats (TIRs) – we next tested for integration of DNA without TIRs.

*A. boonei* casposase randomly integrates into pUC19 a blunt-ended DNA duplex (dsLE30) that contained sequence from the Left End (LE) TIR of the *A. boonei* casposon (LE30) [16]. We used this as a ‘standard’ to compare casposase catalysed integration of dsDNA and ssDNA molecules with and without TIR sequences. Integration of different DNA substrates was identified by incorporation of the Cy5 label into plasmid pACYC-Duet, generating nicked and linearized plasmids consistent with single-end and double-end DNA products (Figure 1B). Casposase integration activity was increased relative to dsLE30 when substrate was dsDNA with 3′-ssDNA overhangs (dsran28+3′, compare Figure 1B lane 5 with 8) that is an effective protospacer for integration by Cas1-Cas2 *in vitro* [10]. The ssDNA molecules LE30-atk, ssran28, ssran50 were also integrated (Figure 1B lanes 4, 7, 9 and Figure 1C), in agreement with the stimulatory effect on integration of 3′ ssDNA overhangs [15,18]. However, LE30-top was not integrated at all and ssran19 was a poor ssDNA substrate – each lacks a cytosine at the -3 position from the 3′ end, a nucleotide that is important for catalysis by *A. boonei* casposase (Figure 1B lanes 3, 6 and Figure 1C) [15,18]. DNA integration by casposase *in vitro* was not inhibited if the ssDNA was pre-bound with *E. coli* Single-Strand DNA Binding protein (SSB, Supplementary Figure S4), indicating that casposase is proficient at binding to DNA ends of a nucleoprotein filament.

We next tested if *A. boonei* casposase can integrate the β-lactamase gene (DNA-*bla*) into pACYC-Duet when flanked with TIRs or not. To determine if full site integration of DNA-*bla* had occurred, and had retained functional ampicillin resistance, integration products were ethanol precipitated and transformed into *E. coli* cells. Plasmids purified from chloramphenicol-ampicillin (chlm-amp) agar were restriction digested to verify the insertion of DNA-*bla* with TIRs (mini-casposon), or without TIRs (Supplementary Figure S5). An estimate of the efficiency of DNA-*bla* integration was made from colony counts from chloramphenicol agar, which selects for pACYC-Duet, and from chlm-amp agar for successful double-end integrations – full data in Supplementary Figure S6. This gave efficiencies of 9.48 × 10^−6^ for the TIR containing DNA-*bla* and 1.7910^−6^ for DNA-*bla* that lacked TIR (Figure 1D). Sequencing of plasmid DNA purified from chlm-amp agar colonies confirmed the integration of TIR or non-TIR flanking long DNA substrates. Sequencing results also confirmed that integration had caused a 15 base-pair sequence duplication, as observed from integration of a TIR-flanked gene before [16,17] (Supplementary Figure S7A,B). Although a recent study [15,18] and our result (Figure 1C) showed the nucleophilic attack strand of DNA substrates required a C in the terminal -3 position, DNA-*bla* without TIRs does not have a C in the -3 position at both ends and it was successfully integrated by casposase (Supplementary Figure S7C). From 11 sequenced DNA-*bla* integrated pACYC-Duet, it was observed that the long DNA substrate was integrated into different locations (Figure 1E). A DNA logo plot revealed that the first nucleotide of the target site (nucleotide 16 in the DNA logo plot) and the second nucleotide upstream of the target site (nucleotide 14) were preferentially either a G or a T (Figure 1F). These integration sites did not strictly conform to the upstream and target sequence identified in the *A. boonei* casposase preferred target, tRNA-Pro gene [15,18]. These assays highlight the substrate flexibility of *A. boonei* casposase for DNA integration into different sites of pACYC-Duet. It led us to investigate if these characteristics could be utilised for site-specific DNA insertion targeted by Cas9 R-loop formation.

### Engineering of a catalytically active Casposase-Cas9 fusion protein

**Figure 2:**
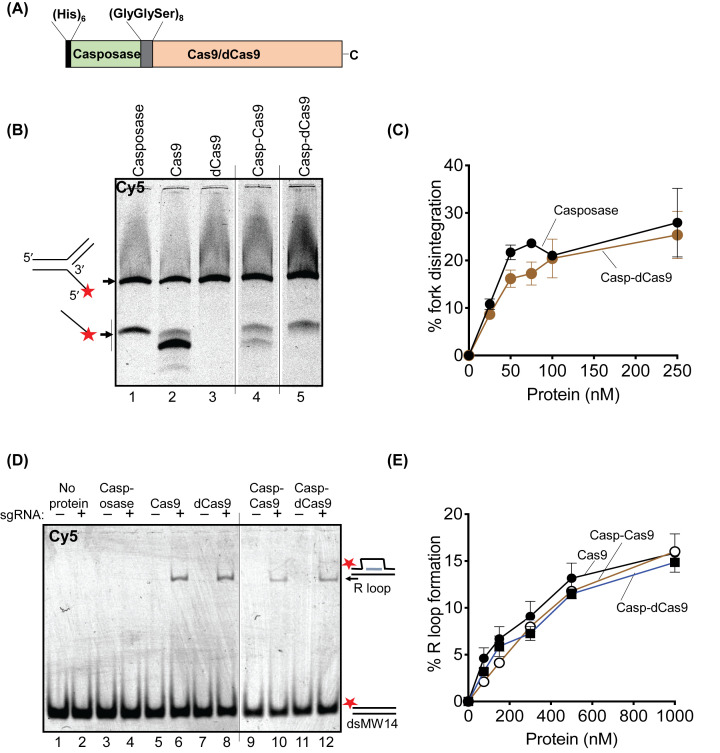
Casp–Cas9 and Casp–dCas9 engineered fusion proteins are catalytically active *in vitro* (**A**) The scheme illustrates the organisation of Casp-Cas9/dCas9 fusion proteins used in this work. (**B**) DNA fork disintegration assay to detect casposase-catalysed nucleophilic attack from a 3′-OH to remove a 5′ ssDNA flap, a reaction indicated to the left of the gel panel. Casposase activity of Casp–Cas9 and Casp-dCas9 (lanes 4 and 5) are compared with casposase, Cas9 and dCas9 (lanes 1–3). All proteins were used at 150 nM and incubated with 25 nM of DNA fork-3 for 1 h. The image detects the Cy5-end label (red star) on a single denaturing acrylamide gel – irrelevant lanes to this work (containing non-functional Casp–Cas9 fusions) are removed from the image, as indicated by the black lines between lanes 3-5. (**C**) Quantification of disintegration activity of Casp-dCas9 compared with casposase, proteins at 0, 25, 50, 75, 100, 250 nM, and fork-3 at 25 nM – a representative gel is given in Supplementary Figure S8. Error bars represent standard error of the mean. (**D**) EMSA of R-loop formation between sgran50 (300 nM) and dsran50 DNA (25 nM) catalysed by Casp–Cas9/dCas9 compared to Cas9/dCas9 controls (all proteins at 150 nM). Nucleic acids were migrated through TBE acrylamide gels prior to imaging to detect the position of Cy5 from dsran50. The image is taken from a single gel, but with irrelevant lanes removed as indicated by the black line between lanes 8 and 9. (**E**) Quantification of R-loop formation by Cas9 and Casp–Cas9/dCas9 using the same assay as in part (**E**), but proteins at 0, 75, 150, 300, 500 and 1000 nM – a representative gel of this assay is given in Supplementary Figure S10A. Error bars represent standard error of the mean.

We aimed to test casposase for RNA-guided integration of diverse DNA substrates by creating fusion proteins with *Streptococcus pyogenes* Cas9. Fusions contained active Cas9, which cuts DNA with a single double-strand break at sites where it forms an R-loop (Casp–Cas9), or dCas9 that is inactivated for nuclease activity by the double amino acid substitutions D10A and H840A (Casp–dCas9) (Figure 2A). Casp–Cas9/dCas9 were stably expressed in *E. coli* when a (Gly-Gly-Ser, GGS)_8_ amino acid linker was engineered between the two proteins. We first established that rudimentary catalytic properties of the casposase and Cas9/dCas9 moieties were retained within purified fusion proteins (Supplementary Figure S2B), by measuring casposase-catalysed ‘disintegration’ and Cas9-catalysed R-loop formation (Figure 2B–E). The casposase product from fork-3 (Figures 1A and 2B, lane 1) was also observed from Casp–Cas9 and Casp–dCas9 (Figure 2B, lanes 4 and 5). Visualising DNA products from Casp-Cas9 (lane 4) was complicated by the fact that Cas9 alone cut the fork-3 substrate (lane 2). However, casposase was active in the fusion proteins evident from comparing catalytically inactive dCas9 (lane 3), with Casp–dCas9 (lane 5) that gave the same single product as casposase alone. Comparison of ‘disintegration’ activity on fork-3 over a range of protein concentrations further confirmed functional casposase activity from Casp-dCas9 (Figure 2C and Supplementary Figure S8).

R-loop formation was assessed by providing Casp–Cas9/dCas9 with sgRNA. R-loop formation between sgran50 and dsran50 DNA by Casp–Cas9 or Casp–dCas9 – the dsran50 DNA target contains the PAM 5′-TGG – was readily detected after de-proteinising the reactions (Figure 2D, lanes 6–12). These were confirmed to be *bona fide* R-loops by treatment with RNaseH (Supplementary Figure S9), which degrades RNA when hybridised to DNA. Efficiency of R-loop formation was similar for Cas9, Casp–Cas9 and Casp–dCas9 measured over a range of protein concentrations (Figure 2E and Supplementary Figure S10A). Although Casp–Cas9/dCas9 formed R-loops, in EMSAs that were not de-proteinised we observed significantly altered migration of protein–DNA binding complexes from Casp–dCas9 and Casp–Cas9, compared with Cas9 or dCas9, including substantial ‘in-well’ complexes from the fusion proteins (Supplementary Figure S10B). These differences cannot be explained solely by differing molecular masses of monomeric Cas9/dCas9 and Casp–Cas9/dCas9 (160 and 209 kDa, respectively), but may be caused because casposase tetramerises on DNA [15] that might result in very large EMSA complexes observable as ‘in-well’ aggregates. Additionally, in the fusion proteins the Cas9 and casposase moieties are likely to compete for DNA binding at multiple sites giving the observed EMSAs, though alleviated by sequence specificity from sgRNA. Nevertheless, Casp–Cas9/dCas9 were catalytically active as fusion proteins, and we therefore next tested DNA integration activities.

### RNA-guided DNA integration by Casposase–Cas9

**Figure 3:**
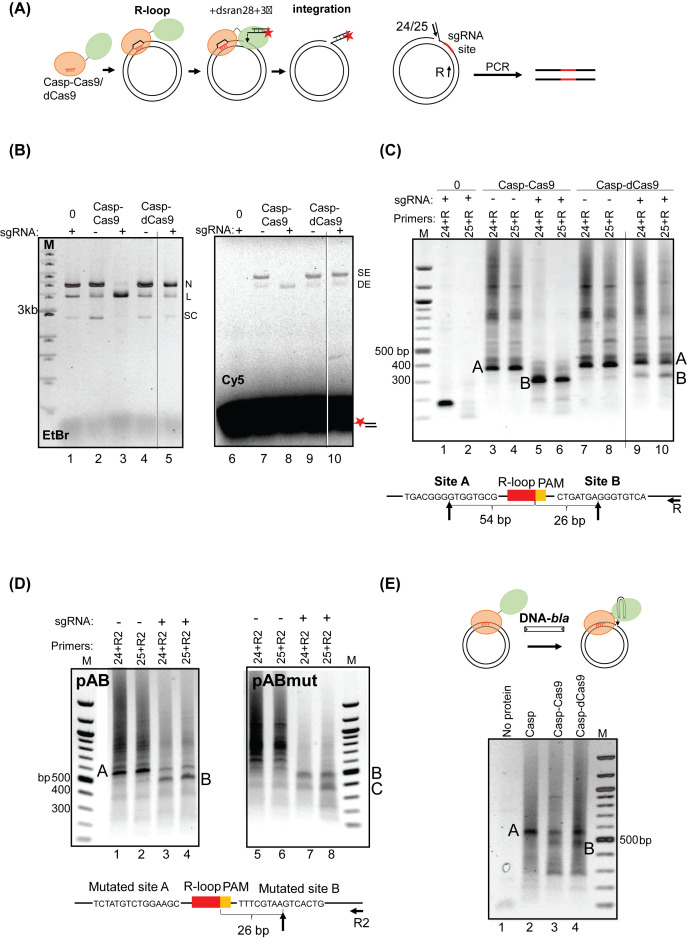
DNA integration by Casp-Cas9/dCas9 was targeted by R-loop formation (**A**) Representative diagrams illustrate the stepwise integration assay (left) and PCR reaction used to detect substrate integration (right). (**B**) Assays for R-loop guided integration of dsran28+3′ into pACYC-Duet were assessed using ethidium bromide (EtBr) to indicate supercoiled (SC), linearized (L) and nicked (N) plasmid, and fluorescence to detect Cy5-end labelled dsran28+3′ (‘Cy5’) that was single end (SE) or double end (DE) integrated. Reactions lasted 1 h and contained 300 nM sgACYC as indicated, and either no protein (‘0’) or 150 nM of Casp-Cas9 or Casp-dCas9 as indicated. A single gel is shown, with lanes removed that are irrelevant to this panel, indicated by the thin vertical black line. (**C**) Plasmid DNA products from (**B**) were gel extracted and PCR amplified to determine sites of dsran28+3′ integration. PCR reactions used primers 24 or 25 to anneal integrated DNA, plus primer R to anneal pACYC-Duet as shown in (A). Site A and site B DNA bands were gel extracted and sequenced, giving integration sites shown below the gel panels. A single gel is shown, with lanes removed, indicated by the thin vertical black line. The band visible in lane 1 was due to non-specific binding of the primer 24 to the plasmid in the absence of integration reaction. (**D**) RNA-guided (sgACYC) integration of dsran28+3′ by 150 nM Casp-Cas9 was assessed into sites A and B in pAB, or into the plasmid pABmut in which sites A and B were mutated. PCR reactions used primers 24 or 25 to anneal integrated DNA, plus primer R2 to anneal the pMA-T backbone. When pABmut was used integration was lost at site A but was retained at site B when sgRNA was used, (lanes 5-8) − DNA sequencing confirmed that site B was again 26 bp downstream of PAM despite the change in nucleotide sequence. Also labelled (‘C’) is the additional new product referred to in the results. (**E**) Ethidium bromide stained agarose gel of PCR amplification of DNA-*bla*-TIR integration catalysed by casposase and fusion proteins. The major PCR products were sequenced − as indicated on the gel, the slower migrating band corresponded to integration at site A, and the faster band to site B integration.

*A. boonei* casposase integrates DNA into different locations of pACYC-Duet lacking the tRNA-Pro sequences it targets in nature [16]. We tested if Casp–Cas9/dCas9 integrated dsran28+3′ DNA, and if integration could be targeted to an R-loop formed from a sgRNA. The sgRNA (sgACYC) targeted sequence next to a 5′-TGG PAM close to the pACYC-Duet p15A replication origin – the step-wise reaction is summarised in Figure 3A. Ethidium bromide was used to see plasmid DNA products (Figure 3B, left) confirming that R-loop targeting was effective because Casp–sgACYC–Cas9 gave linearized plasmid (lane 3), but did not when sgACYC was absent (lane 2). Casp–dCas9 did not linearize the plasmid either with or without sgACYC because dCas9 cannot make DNA double-strand breaks (lanes 4 and 5).

Imaging the Cy5 label (Figure 3B, right) showed that Casp–Cas9 and Casp–dCas9 integrated dsran28+3′ DNA into the plasmid forming single-end and double-end (nicked and linearized) products, either in the presence or absence of sgRNA (Figure 3B, lanes 6–10). DNA integration was abolished when the casposase moiety of fusion proteins was inactivated by mutation D254A (Supplementary Figure S11). Therefore, fusion proteins retained the non-specific integration activity expected of casposase, which in these gels is indistinguishable from any sgRNA targeted integration. We analysed reaction products to assess if sgACYC was targeting integration by casposase. DNA integration sites were first established by PCR amplification of reaction products using a primer annealing to integrated dsran28+3′ DNA (we used primers annealing to both strands of the duplex, ‘24’ and ‘25’) and a primer (‘R’) annealing to pACYC-Duet close to the sgACYC target site – summarised in Figure 3A,C. DNA integration in reactions without sgACYC gave a wide range of PCR products indicating integration into different locations, but also an unexpected ‘hot-spot’ product of 350–400 base pairs, referred to as ‘site A’ (Figure 3C, lanes 3 and 4). Reactions containing sgACYC resulted in a concentration of PCR products at 300 base pairs, consistent with what is expected for successful targeting by sgRNA (Figure 3C, lanes 5 and 6), referred to as ‘site B’. Plasmid DNA strand breakage by Cas9 during DNA integration by Casp–sgACYC–Cas9 would halt chain extension at the break site in subsequent PCR, giving increased readout of this PCR product relative to any other products. Significantly, however, Casp–sgACYC–dCas9, which cannot break DNA, also gave substantially more ‘site B’ PCR product (Figure 3C, lanes 9 and 10) than when sgACYC was absent (lanes 7 and 8). DNA sequencing of the PCR products indicated that Casp–sgACYC–Cas9/dCas9 integrated dsran28+3′ into site B located 26 base-pairs from the R-loop target site, and that the unexpected site A was located 80 base pairs away from site B, 54 base pairs from the R-loop (Figure 3C). Interestingly, site A was also one of the 11 sequenced integration sites in the long DNA substrate integration catalysed by casposase. Therefore, the upstream and target sequence of site A largely conformed to the sequence logo reported in Figure 1F and Supplementary Figure S12. Site B did not conform to the sequence logo at the upstream-target site border and both sites showed a large difference from the A. boonei preferred target, tRNA-Pro gene.

To confirm that integration into site B was targeted by the sgRNA, rather than by cryptic sequence preference when sgRNA is absent, we mutated the DNA sequence around site B in the target plasmid. To do this we made a new plasmid (pAB) derived from pMA-T comprising 250 bp of synthesised DNA sequence from pACYC-Duet identical to sites A and B, to compare with a plasmid (pABmut) that was identical to pAB except that DNA sequence of sites A and B were randomly mutated over 15 bp. Integration of dsran28+3′ by Casp-Cas9 was compared in the presence and absence of sgACYC (Figure 3D). In reactions targeting unmutated plasmid (pAB), and as expected from Figure 3C, the site A integration product of Casp–Cas9 was predominant in the absence of sgACYC but site B product was the major product when sgACYC was included (Figure 3D compare lanes 1, 2 with lanes 3, 4). The same reactions using the pABmut target resulted in loss of site A product, but site B product was retained when sgACYC was included (Figure 3D, lanes 5–8) and confirmed to be at 26 base-pairs from the PAM. These reactions also generated an additional new product ‘C’, visible in all lanes in Figure 3D, but we determined that this product is artefactual to the PCR reaction, not from integration, because its DNA sequence did not match any sequence of the target plasmid.

These results show that sgRNA is responsible for targeting of DNA integration into site B, and that integration to site A is likely to be caused by fortuitous targeting from casposase enzyme. We conclude that casposase-catalysed DNA integration can be targeted by sgRNA when tethered to Cas9 or dCas9, although random integration from the casposase moiety of Casp–dCas9 also remains active.

### RNA guided gene integration by Casposase–Cas9 fusion protein

We next tested if integration of the gene *bla* could also be guided to target site B by sgACYC. We incubated 150 nM of protein-sgACYC with 150 ng of pACYC-Duet and equimolar DNA-*bla*-TIR (mini-casposon, 45 ng). Target plasmid was ethanol precipitated for use as a PCR template to detect DNA integration (Figure 3E). As expected, in the absence of protein (lane 1) there was no detectable DNA integration, and casposase gave the approximately 550 bp product, including sgACYC for Casp–Cas9 and Casp–dCas9 gave an additional band at approximately 480 bp (lanes 3 and 4), which DNA sequencing identified as being at site B, as observed for dsran28+3′ in Figure 3C. Sequencing also confirmed that the 550 bp product corresponded to ‘site A’ integration in the absence of sgACYC. We tried to determine if integration products from DNA-*bla* into pACYC-Duet gave intact gene sequence that could confer ampicillin resistance after transformation into *E. coli* DH5α. We were able to recover a few ampicillin resistant colonies, but each showed off-target random integration sites within the plasmid rather than at site B. This is discussed more below, but our inability to recover colonies from site B integration products may indicate that this causes irreparable damage to pACYC-Duet that makes it unable to transform, or to be defective in cells. We therefore next assessed if Casp–Cas9/dCas9 was able to find its target DNA effectively when expressed in *E. coli* cells.

### RNA guided inactivation of *lacZ* by expression of Casposase–Cas9 in *E. coli*

**Figure 4:**
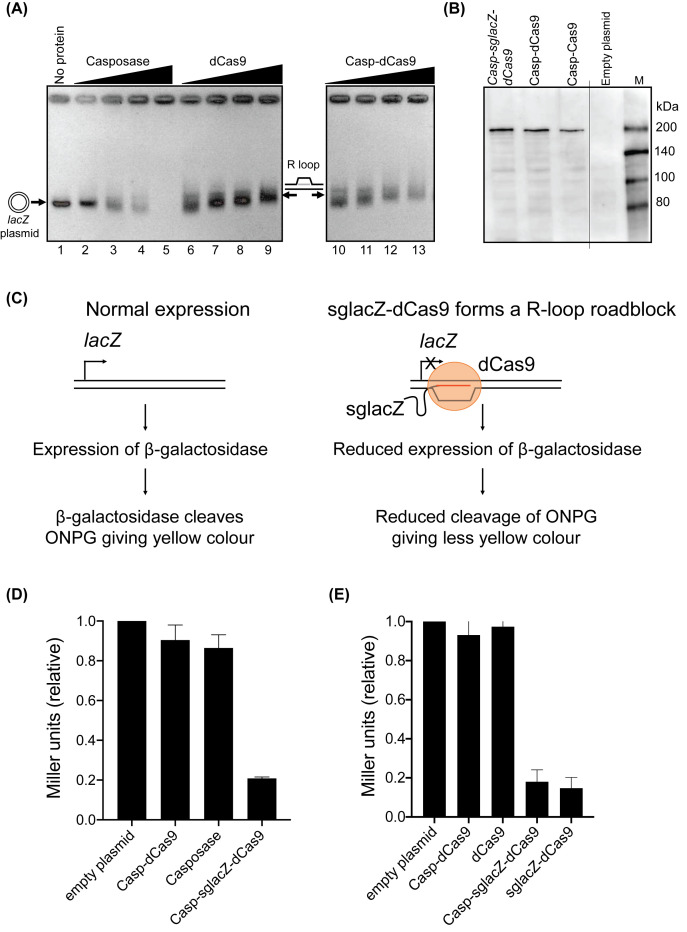
Casp–dCas9 targeted *lacZ* inside *E. coli* cells (**A**) *In vitro* R-loop formation shown in an ethidium bromide stained agarose gel using sg*lac*Z targeting of pUC19 (150 ng). Proteins were as indicated at 40, 75, 150 and 300 nM, or with no protein in lane 1 – casposase (lanes 2–5), dCas9 (lanes 6–9) and Casp–dCas9 (lanes 10–13). Reactions were incubated at 37°C for 20 min. (**B**) A Western blot using anti-(His)_6_ antibody confirmed that the Casp–Cas9/dCas9 protein were stably expressed for *lacZ* targeting in *E. coli* MG1655 K-12 Δ*lacZ*::T7 RNAP cells transformed with pRC7 and plasmids for expressing proteins and sg*lacZ*. A single gel is shown, with a blank lane removed, indicated by the thin vertical black line. The uncropped image was shown in Supplementary Figure S12. (**C**). Schematic illustration of the Miller assay and the effect of sglacZ-dCas9 on the Miller assay. Miller assay measures the activity of β-galactosidase that cleaves ONPG to yield o-nitrophenol, which has a yellow color. sglacZ-dCas9 forms a R-loop at the transcription start site in the *lacZ* gene, thus reducing β-galactosidase expression and the Miller unit. (**D** and **E**) Miller assays to measure Casp–dCas9 targeting of the *lacZ* gene on (D) plasmid pRC7, or (E) the chromosome. Miller units were normalised to control ‘empty plasmid’ that lacks Casp–dCas9 or casposase encoding genes.

To test for R-loop formation in *E. coli* cells we used Casp–dCas9 to avoid DNA breaks triggering SOS reponses and cell death associated with expression of active Cas9 [31]. A sgRNA targeting *lacZ* (sglacZ) mixed with Casp–dCas9 was confirmed to be effective for R-loop formation against plasmid pUC19 *in vitro* (Figure 4A). Casp–dCas9 with or without sglacZ was expressed in CL003, *E. coli* MG1655 K-12 Δ*lacZ* cells, which had been further modified to inducibly produce T7 RNA polymerase for temporal control of Casp–dCas9 expression (Figure 4B; Supplementary Figure S13 and Table S4). Cells were transformed with pRC7, a very low copy number plasmid containing the *lacZ* gene and assayed for β-galactosidase activity as a read-out for Casp–sglacZ–dCas9 targeting compared to controls (Figure 4C,D). Measurements took into account potential differences in live cell numbers by verifying parity through viability colony counts (Supplementary Figure S14A,B). Cells expressing Casp–sglacZ–dCas9 showed five-fold reduced β-galactosidase activity compared with cells expressing Casp–dCas9 without sglacZ. We observed a similar effect of Casp–sglacZ–dCas9 against a chromosomal *lacZ* gene when repeating the β-galactosidase assay from *E. coli* BL21 AI cells (Figure 4E). Therefore Casp–sgRNA–dCas9 is effective for targeting *lacZ* in these cells, and we next extended the experiment by electroporating linear DNA into cells expressing Casp–sglacZ–dCas9 to assess whether DNA insertion could be detected into lacZ. However, no white colonies were observed when cells were plated out onto LB agar containing X-gal, indicating that *lacZ* had not been disrupted by DNA insertion via casposase-catalyzed DNA integration. Further analysis showed that the integration activity of casposase might be reduced *in vivo*, illustrated by *in vitro* DNA integration in the presence of human cell free extract (CFE) and Mg^2+^ (Supplementary Figure S15A,B).

## Discussion

Casposase enzymes are thought to be antecedents of Cas1 integrases that underpin CRISPR immunity [14]. Recent structural analysis of a casposase bound to DNA [15] highlighted how elements of its protein architecture might have evolved into Cas1, in particular by accommodating interaction with Cas2, forming the means for capture and integration of protospacer DNA into CRISPR loci. Casposases also target integration to specific DNA sites – the tRNA-Pro gene [17], the tRNA-Leu gene [15], or the gene encoding protein EF2 [15,18] – but they integrate DNA into different sites in the absence of a target. A previous study also generated a DNA logo blot from 20 mini-casposon integration sites in pUC19 catalysed by *A. boonei* casposase [16]. This DNA logo plot showed no pattern of the nucleotide frequency at each position, whereas our DNA logo plot from 10 mini-casposon integration sites in pACYC-Duet showed a G or T is frequently found at the upstream-target site border. In addition, these integration sites did not resemble the natural upstream sequence and target sequence in *A. boonei* casposon. The amplification of integrated plasmids catalysed by fusion proteins in the absence of sgRNA illustrated a specific band pattern instead of DNA smearing and this suggests casposase mediated integration is not completely random in the absence of its native target. To better understand the sequence motif at the upstream-target site border recognised by casposase, a larger sample size of long DNA integration will be needed to draw a more accurate conclusion. In agreement with two recent reports [15], we observed considerable substrate flexibility for integration by casposase, and a preference for ssDNA located at ends containing a cytosine residue located at the -3 position from the DNA 3′ end. We report that *A. boonei* casposase integrates a variety of DNA molecules, including functional β-lactamase gene, without flanking TIRs. This supports the idea that Cas1 evolved from casposases [15,18]. During evolutionary transformation from casposase to CRISPR Cas1, Cas1 enhanced the promiscuous DNA substrate integrating property and recruited Cas2 to fix the integrating DNA length.

The substrate flexibility of DNA integration by casposase seemed an attractive proposition for testing if it could be targeted to specific DNA sites by a guide RNA, if casposase were fused to Cas9. Several attempts have been made to target transposons for delivery of defined DNA substrate, including fusions to Cas9 [22–25]. These offer potential for genetic editing reactions, by insertion of user-defined DNA sequences into user-defined target sites without the need for homology-directed DNA repair. We isolated a casposase fusion to Cas9 or dCas9 (Casp–Cas9/dCas9) that retained the expected catalytic activities, including the ability to integrate DNA without TIRs. Our *in vitro* data indicated DNA integration could be RNA guided to a site (site B) located 26 base-pairs from the Cas9 PAM sequence. Integration of a 28 base-pair (dsran28+3′) or 1200 base pair (DNA-*bla*) duplex into site B was significant only when the sgRNA was present, but in both cases additional products of integration were present at other sites. This is therefore encouraging as a prototype for further studies, because it indicates achievable targeting of DNA integration by Casp–Cas9/dCas9. It was significant that DNA integration was proficient in the Casp–dCas9 fusion, because it is advantageous to avoid potentially toxic DNA strand breaks caused by active Cas9 [32].

The stringency of the Casp–Cas9/dCas9 targeting requires improvement to reduce ‘off-target’ integration. The off-target integration products most likely to arise from misguided integration from unguided casposase activity, rather than off-target R-loop formation that is a well-known problem of CRISPR editing reactions. We think this because fusion of casposase to ‘high-fidelity’ HF-Cas9 [33] gave similar results *in vitro* to those of Casp–Cas9/dCas9 (not shown). The casposase moiety of Casp–Cas9/dCas9 is likely to compete with the Cas9–sgRNA moiety for DNA binding, given that casposase is proficient at random DNA integration. It may be possible to reduce or overcome this by eliminating residual amounts of ‘free’ casposase that can be detected in purified preparations of Casp–Cas9/dCas9, and by temporal control of the delivery of fused Casp–Cas9/dCas9 *in vitro*, or during expression inside cells. By using the recently available crystal structure of a casposase [15], efforts are underway to engineer *A. boonei* casposase to show reduced DNA binding affinity in anisotropy measurements whilst retaining the ability to integrate DNA.

In this work we also identified that Casp–Cas9/Casp–dCas9 can successfully target the *lacZ* gene in an *E. coli* chromosome or a plasmid, deduced from reduced β-galactosidase activity in cell populations measured in Miller assays. This indicated reduced transcription of *lacZ* that can be attributed to Casp–dCas9 being a ‘roadblock’ as observed for dCas9 alone. However, there was no evidence for Casp-dCas9-catalysed DNA insertion at the *lacZ* target when linearized DNA was electroporated into cells – no white colonies were detected on Xgal-agar screening. This may result from rapid degradation of linear DNA inside *E. coli* cells with active RecBCD [34]. Casposase was able to integrate DNA oligonucleotides *in vitro* in the presence of human cell-free extract and Mg^2+^, albeit with lower efficiency. The ability of Casp–Cas9/dCas9 to achieve RNA-targeted DNA integration will be further assessed in human cells.

## Conclusions

*A. boonei* casposase does not strictly rely on TIRs for integration and can integrate random sequences, especially for short oligonucleotides with 3′ overhangs. This substrate flexibility of casposase might be useful in targeted DNA insertion. We demonstrate the casposase and Cas9 moieties of a fusion protein were both active. Fusion proteins were capable of integrating DNA substrates without TIRs and forming R-loop with target DNA in the presence of sgRNA. On top of these, it was observed that fusion protein-catalysed DNA integration could be guided by sgRNA to a site 26 bp from the Cas9 PAM. We conclude from this work that Casp–dCas9 can catalyse RNA-guided DNA integration and is a useful prototype for refinement of *in vitro* activities for testing in cells.

## Supplementary Material

Supplementary Figures S1-15- Tables S1-S4

## Abbreviations

HR: homologous recombination
MGE: mobile genetic element
TIR: terminal inverted repeat
