# Supplementary material for: Integration of diverse DNA substrates by a casposase can be targeted to R-loops in vitro by its fusion to Cas9
Source: Biosci Rep. 2021 Jan 5;41(1):BSR20203595. doi: 10.1042/BSR20203595 (PMC7786333; doi:10.1042/BSR20203595)

Supplementary Table 1. Plasmids used.

Name	Comment
**pCL2**	*A. Boonei* Casposase in pACYC-Duet
**pCL3**	Casposase-$(GGS)_8$-Cas9 (Casp-Cas9) in pACYC-Duet
**pCL4**	Casposase-$(GGS)_8$-dCas9 (Casp-dCas9) in pACYC-Duet
**pAB**	GeneArt plasmid containing 250bp from pACYC-Duet that flanked site A and B
**pABmut**	Same as pAB except both 15bp flanking site A and B were mutated into random sequences
**pCaspamp**	*amp*^{R} gene in pET14b was flanked with terminal inverted repeats from *A. boonei* casposon
**pET14b**	Used to amplify the gene *bla* for generating DNA-*bla* integration substrates with and without TIRs.
**pglacZ+**	sgRNA targeting to + strand of *E. coli lacZ* gene in pCDF-1b
**pglacZ-**	sgRNA targeting to - strand of *E. coli lacZ* gene in pCDF-1b
**pCL5**	Casp-Cas9, sgRNA targeting to - strand of *E. coli lacZ* gene in pACYC-Duet
**pCL6**	Casp-dCas9, sgRNA targeting to – strand of *E. coli lacZ* gene in pACYC-DUet
**pUC19**	Used for in vitro R-loop formation with plasmid assay
**pRC7**	pRC7 contains *lac* operon, it was used for Miller assay to detect in vivo targeted binding of fusion proteins.

Supplementary Table 2. DNA primers used - The lower-case sequence are non-annealing 'overhangs' used for molecular cloning.

Name	Sequence (5' to 3')
**Casp pACYC F**	gatggatcctATGAACCCTCTTTTAGTTAGTGG
**Casp pACYC R**	gccctgcagCTATTTTAATTTACTCTTTACCTTCCC
**Cas9 pACYC F**	ggccggctgcagATGGATAAGAAATACTCAATAGGCTTAG
**Cas9 pACYC R**	taaatgcggccgcTCAGTCACCTCCTAGCTGA
**Casp-GGS8-Cas9 F**	ggtgggtcaggcggttcgggaggcagtggtggaagcATGGATAAGAAATACTCAATAG
**Casp-GGS8-Cas9 R2**	actaccaccacttccgccagaccctccagatccgccTTTTAATTTACTCTTTACCTTCC
**Casposon LE F**	gggatgtatatatatccccGATAAGCTTTAATGCGGTAG
**Casposon LE R**	ctcttaagttccctttttcaGATGATAAGCTGTCAAACATG
**Casposon RE F**	ctcttaagttcccttttttaTATCAAAAAGGATCTTCACC
**Casposon RE R**	gggatatatatatatccccATCTCATGACCAAAATCC
**Casp Amp F**	ggggatatatatacatcccctcttaagttccctttttcaGATGATAAGC
**Casp Amp R**	ggggatatatatatatcccctcttaagttcccttttttaTATCAAAAAGGATCTTCACC
**AmpR F**	TTCTTAGACGTCAGGTGGCAC
**AmpR R**	TATTAGACGTCGAGTAAACTTGGTCTGACAGTTACC
**SPIN F**	GCGGAGCCTATGGAAAAACG
**AmpRE**	ATGGTAAGCCCTCCCGTATC
**T7 F**	TAATACGACTCACTATAGG
**sgRNA R**	AAGCACCGACTCGGT
**24**	GCAGTCCCCTCGCCTCAGCTACGCTCGT
**25**	CGTAGCTGAGGCGAGGGGACTGCTGGGC
**R**	CGTGATGCTTGTCAGGGGGGCGGAGCCTATGGAAAAACG
**R2**	GCACGAGGGAGCTTCCAGGGGGAAACGC
**sglacZ+ F**	tgtaaaacgaGTTTTAGAGCTAGAAATAGC
**sglacZ+ R**	acgtcgtgacACTAGTATTATACCTAGGAC
**sglacZ- F**	ttgaaacccaGTTTTAGAGCTAGAAATAGC
**sglacZ- R**	tattggcttcACTAGTATTATACCTAGGAC
**sgRNA F**	attctaCTGCAGCCGAAAAGTGCCACCTGAC
**sgRNA R**	aaactagTCTAGACTCGAGTAAGGATCCAG

Supplementary Table 3. DNA oligonucleotides used to generate assay substrates for integration. Nucleotides in red form the T7 promoter for transcription of sgRNA, those in blue are to generate the RNA 'guide' sequences, and in black are nucleotides that form the sgRNA scaffold sequence.

Name	Sequence (5' to 3')
**ssLE30-top (makes dsLE30)**	/Cy5/GGGGATATATATACATCCCCTCTTAAGTTC
**ssLE30-atk (makes dsLE30)**	/Cy5/ GAACTTAAGAGGGGATGTATATATATCCCC
**ssran28 (makes dsran28+3′)**	/Cy5/ GCAGTCCCCTCGCCTCAGCTACGCTCGT
**ssran28 comple (makes dsran28+3′)**	CGTAGCTGAGGCGAGGGGACTGCTGGGC
**ssran50 (makes dsran50 and Fork-3)**	/Cy5/ CAACGTCATAGACGATTACATTGCTACATGGAGCTGTCTAGAGGATCCGA
**MW12 (makes Fork-3)**	TCGGATCCTCTAGACAGCTCCATGATCACTGGCACTGGTAGAATTCGGC
**ssran19**	/Cy5/ TGTAATCGTCTATGACGTT
**PM16 (makes Fork-3)**	TGCCGAATTCTACCAGTGCCAGTGAT
**ssran50 comple (makes dsran50)**	TCGGATCCTCTAGACAGCTCCATGTAGCAATGTAATCGTCTATGACGTTG
**sgran50 ssDNA**	TAATACGACTCACTATAGGTAGACGATTACATTGCTACAG TTTTAGAGCTAGAAATAGCAAGTTAAAATAAGGCTAGTCC GTTATCAACTTGAAAAAGTGGCACCGAGTCGGTGCTT
**sgpACYC ssDNA**	TAATACGACTCACTATAGGAGCGCTAGCGGAGTGTATAC GTTTTAGAGCTAGAAATAGCAAGTTAAAATAAGGCTAGTC CGTTATCAACTTGAAAAAGTGGCACCGAGTCGGTGCTT
**sgpUC19 ssDNA**	TAATACGACTCACTATAGGGTGCTGCAAGGCGATTAAGT GTTTTAGAGCTAGAAATAGCAAGTTAAAATAAGGCTAGTC CGTTATCAACTTGAAAAAGTGGCACCGAGTCGGTGCTT

Supplementary Table 4. *E. coli* strains used. DH5α was used in cloning and for transforming plasmids after integration assays. BL21 A.I. was used in protein expression and purification, and for Miller assays. CL003 and EB377 were used for *in vivo* assays.

Strain	Notable genotype
**DH5α**	F–, ϕ80*lacZ*ΔM15, Δ(*lacZYA-argF*)U169, *rec*A1, *end*A1, *hsd*R17(rK–, mK+), *pho*A, *sup*E44, λ–, *thi*-1, *gyr*A96, *rel*A1
**BL21-AI**	F–, *omp*T, *hsd*SB (rB–, mB–), *gal, dcm, ara*B::T7RNAP-*tet*A
**CL003**	Wild type K-12 MG1655, Δ*lacIYZA::FRT*, *ara*B::T7RNAP-*tet*A
**EB377**	Wild type K-12 MG1655 background, *ara*B::T7RNAP-*tet*A

Figure S1. Substrates used for DNA disintegration and integration assays. See also Figure 1C.

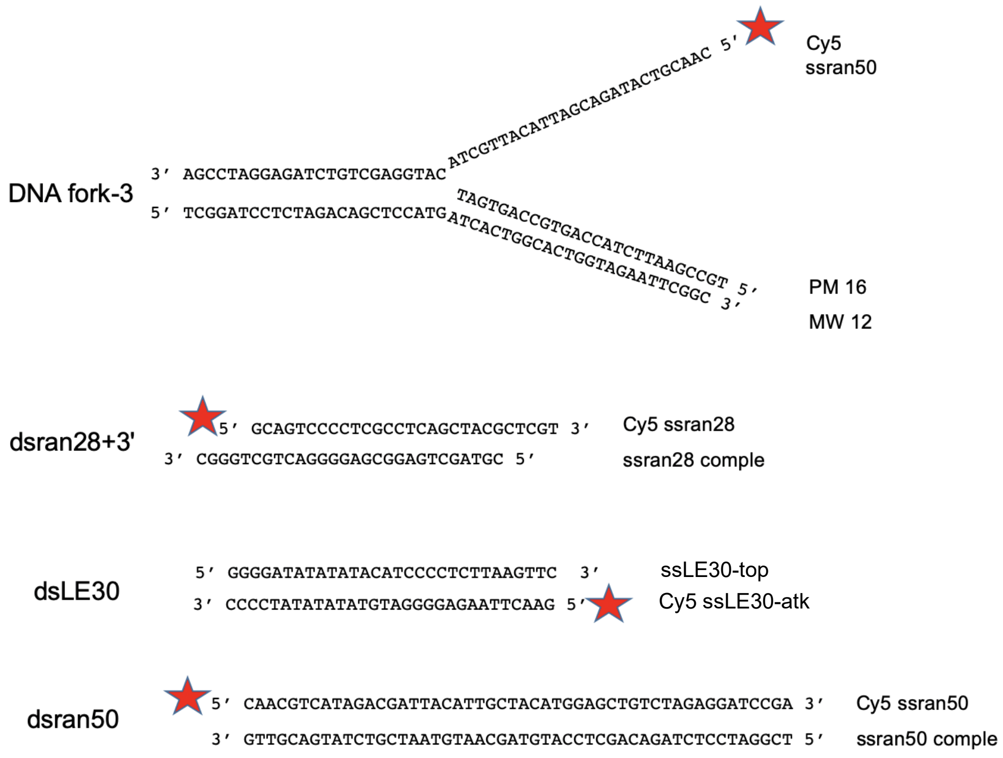

Figure S2 – SDS-PAGE of purified casposase and casposase-Cas9 fusion proteins. (A). Shows summary SDS-PAGE gels for final purified wild-type *A. boonei* casposase and its active site mutants. (B). Summary gel of purified fusions proteins used, in each case loading 5 μg of protein for electrophoresis and coomassie stained. The image is from a single gel but lanes have been moved to remove lanes that were run on the same gel but that are irrelevant to this work.

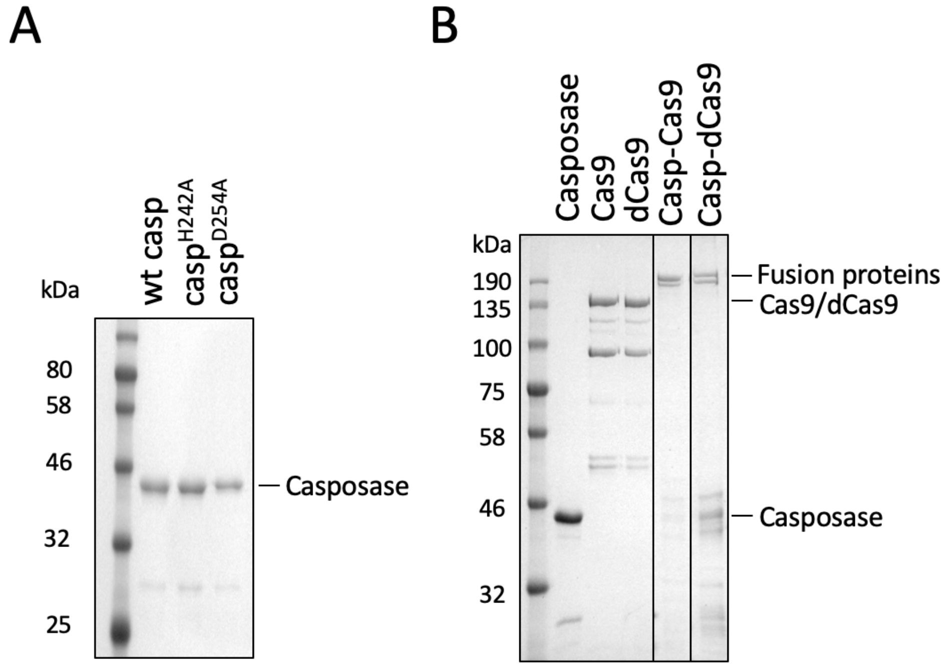

Figure S3. Casposase titration into fork 3 disintegration. Casposase titrated into fork-3 DNA (25 nM) at 0, 50, 75, 100, 125 and 150 nM in reaction buffer containing magnesium or manganese chloride.

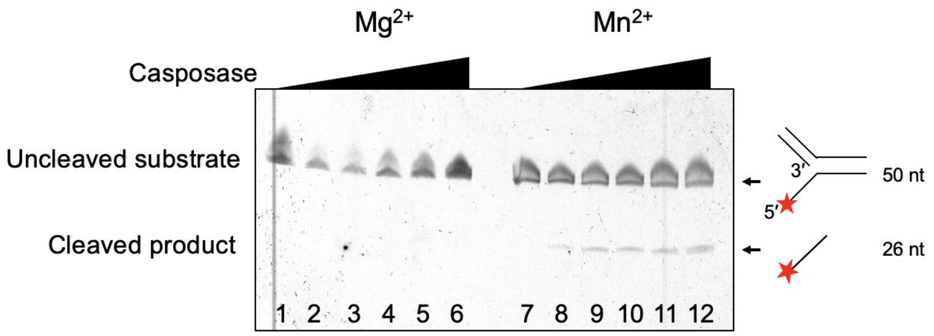

Figure S4. DNA integration is not inhibited by SSB. Reactions contained casposase (0, 20, 40, 75, 150, 300 nM) with 100 nM of ssran28 or 100 nM ssran28 pre-bound with 500 nM SSB (I got the SSB from your freezer) and 150 ng pACYC-Duet. Lanes 7-12 contained 1% SDS added to the proteinase K stop buffer to release ssran28 from SSB.

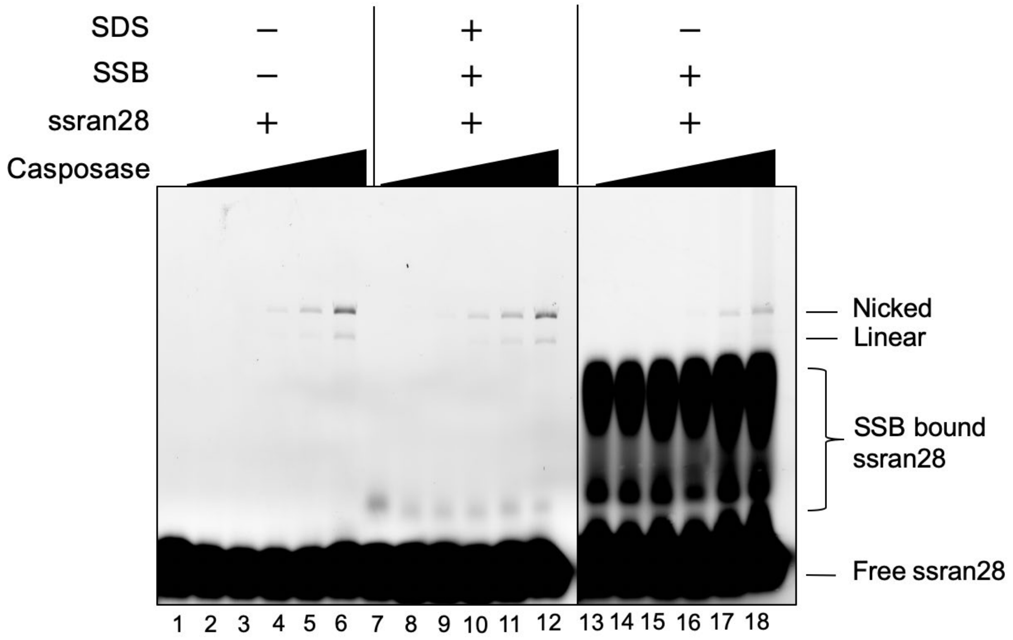

Figure S5. DNA-bla with or without TIRs was inserted into pACYC-Duet by casposase. Long DNA substrates DNA-*bla* with or without TIR were inserted into pACYC-Duet by casposase. After integration reactions, the DNA-*bla* plasmid products were ethanol precipitated and transformed into *E. coli* DH5α. The purified plasmids from chloramphenicol and ampicillin resistant colonies were cut by BamHI to check their size.

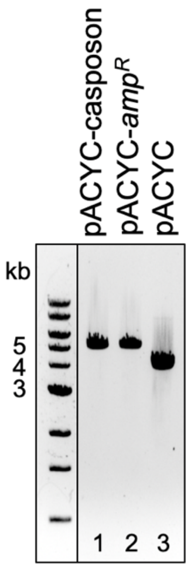

Figure S6. Raw data of transforming DNA-*bla* integrated into pACYC-Duet into *E. coli* DH5α. Transformed cells were spread on chloramphenicol agar to calculate total successfully transformed cells and on chloramphenicol and ampicillin agar to detect DNA-*bla* that has successfully integrated to confer resistance to ampicillin. The colony number on each plate was used to calculate integration efficiency of each long DNA substrate.

Repeat 1:

Long DNA substrates	Colony number on cm plate for 10 µL of cells	Total transformed cells per mL	Colony number on cm and amp plate for 1 mL of cells	Integration efficiency
**mini-casposon**	3200	3.20×10^{5}	4	1.25×10^{-5}
***amp^R* blunt**	6560	6.56×10^{5}	2	3.05×10^{-6}
***amp^R* 3′ overhangs**	6912	6.91×10^{5}	0	0

Repeat 2:

Long DNA substrates	Colony number on cm plate for 10 µL of cells	Total transformed cells per mL	Colony number on cm and amp plate for 1 mL of cells	Integration efficiency
**mini-casposon**	4110	4.11×10^{5}	3	7.30×10^{-6}
***amp^R* blunt**	3500	3.50×10^{5}	0	0
***amp^R* 3′ overhangs**	3810	3.81×10^{5}	0	0

Repeat 3:

Long DNA substrates	Colony number on cm plate for 10 µL of cells	Total transformed cells per mL	Colony number on cm and amp plate for 1 mL of cells	Integration efficiency
**mini-casposon**	3470	3.47×10^{5}	3	8.65×10^{-6}
***amp^R* blunt**	8650	8.65×10^{5}	2	2.31×10^{-6}
***amp^R* 3′ overhangs**	5912	5.91×10^{5}	0	0

Figure S7. Integration of DNA-*bla* into pACYC-duet. Sequencing results verified DNA-*bla* insertion with or without TIR into pACYC-Duet after transformation and purification of the integrated plasmid. After integration reactions, the DNA-*bla* plasmid products were ethanol precipitated and resuspended in dH_2O. The plasmids were transformed into *E. coli* DH5α and purified from ampicillin resistant colonies. The borders at both ends of DNA-*bla*-TIR, mini-casposon, (A) and DNA-*bla* (B) were sequenced to determine the precise integration site and target site duplication (TSD, highlighted region). LE TIR: left end terminal inverted repeat. RE TIR: right end terminal inverted repeat. LE seq and RE seq were sequencing primers used. (C). Comparison of 38 bp terminal sequence at both ends of mini-casposon with that of DNA-*bla*. Only the 3' nucleophilic attack strands are shown.

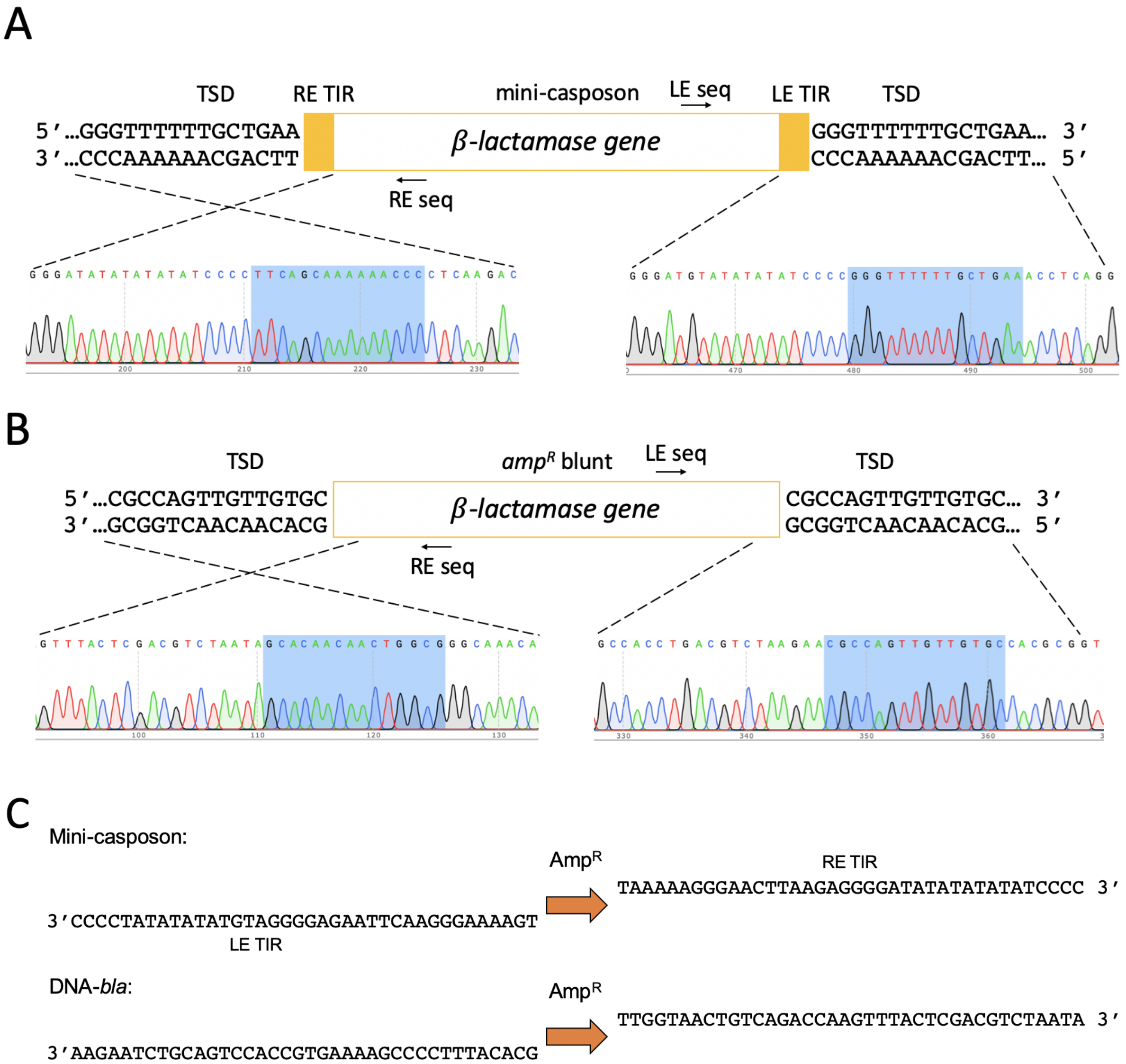

Figure S8. Titration of Casposase and Casp-dCas9 (0, 25, 50, 75, 100, 250 nM) for a disintegration assay. This is a representative gel for quantification in Figure 2C.

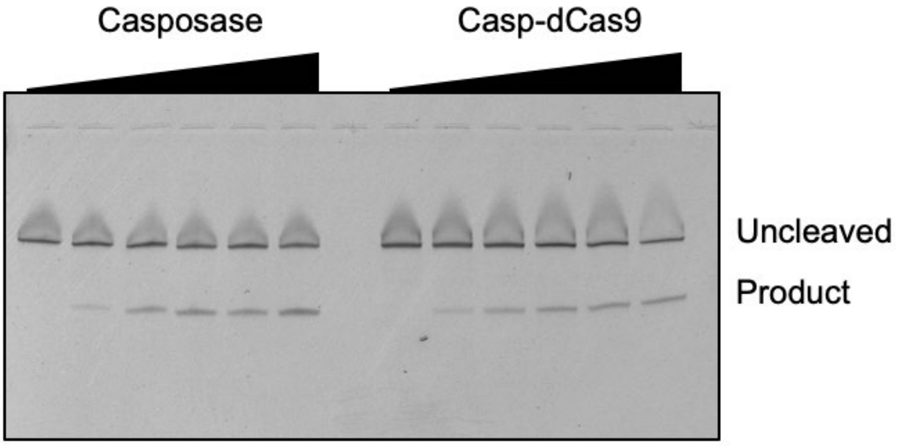

Figure S9. RNase H degraded the R-loop formed between Casp-dCas9-sgran50 and dsran50.

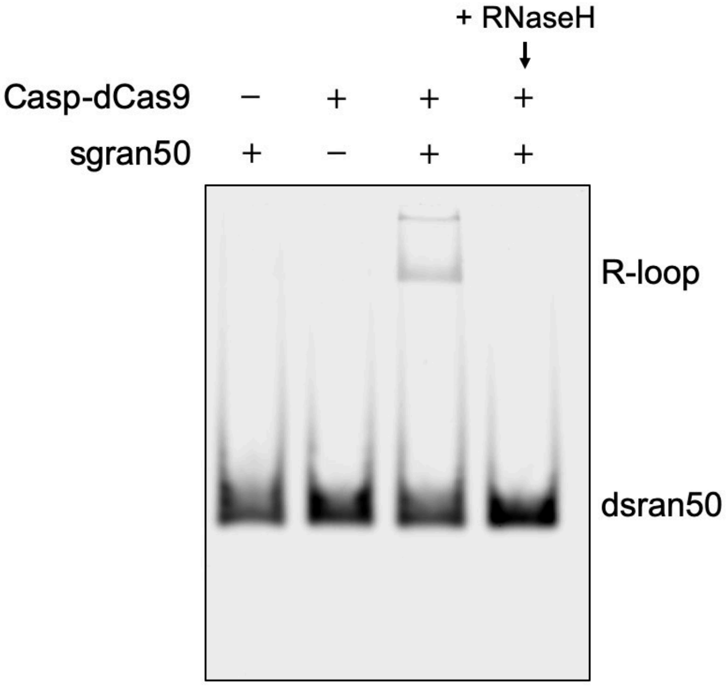

Figure S10. DNA and binding and R-loop formation by proteins at concentrations 75, 150, 300, 500 and 1000 nM. **(A)**. Representative gels showing R-loop formation, used in quantification for Figure 2E. Reactions were treated with proteinase K to detect R-loop formation. **(B).** Cas9 and Casp-Cas9 proteins binding to dsran50 target for R-loop formation, assessed in EMSAs that were not deproteinized – these are representative gels used for DNA binding complex measurements shown in the graph below. Reactions contained 75, 150, 300, 500 and 1000 nM of protein, preincubated with sgran50 before mixing with Cy5 labelled dsran50 (25 nM).

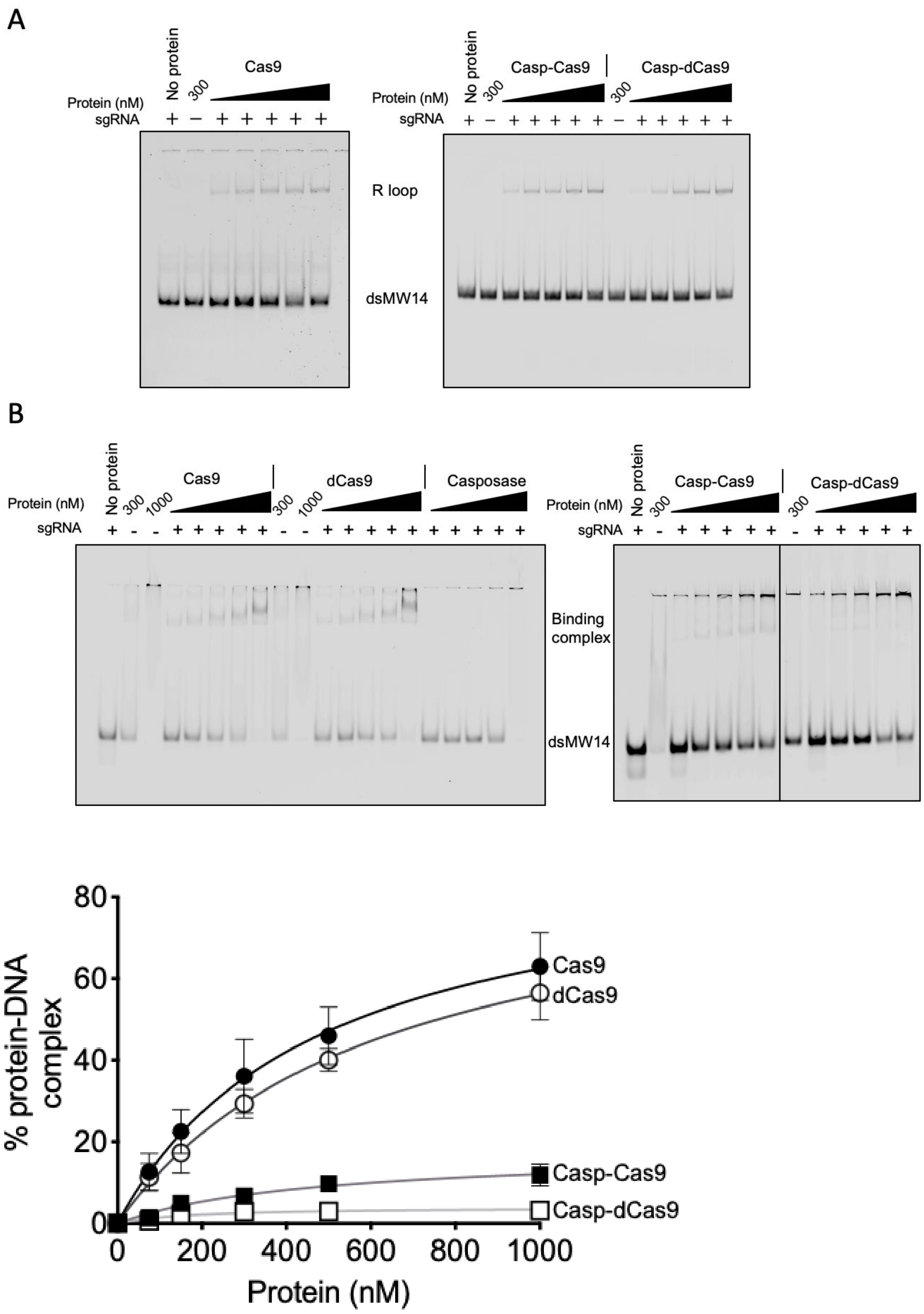

Figure S11. Integration of ssran28 and dsran28+3' into plasmid pACYC-Duet by casposase and Casp-dCas9. The fusion protein containing casposase active site mutant D254A cannot perform integration. The guide RNA (sgACYC) was present in all samples.

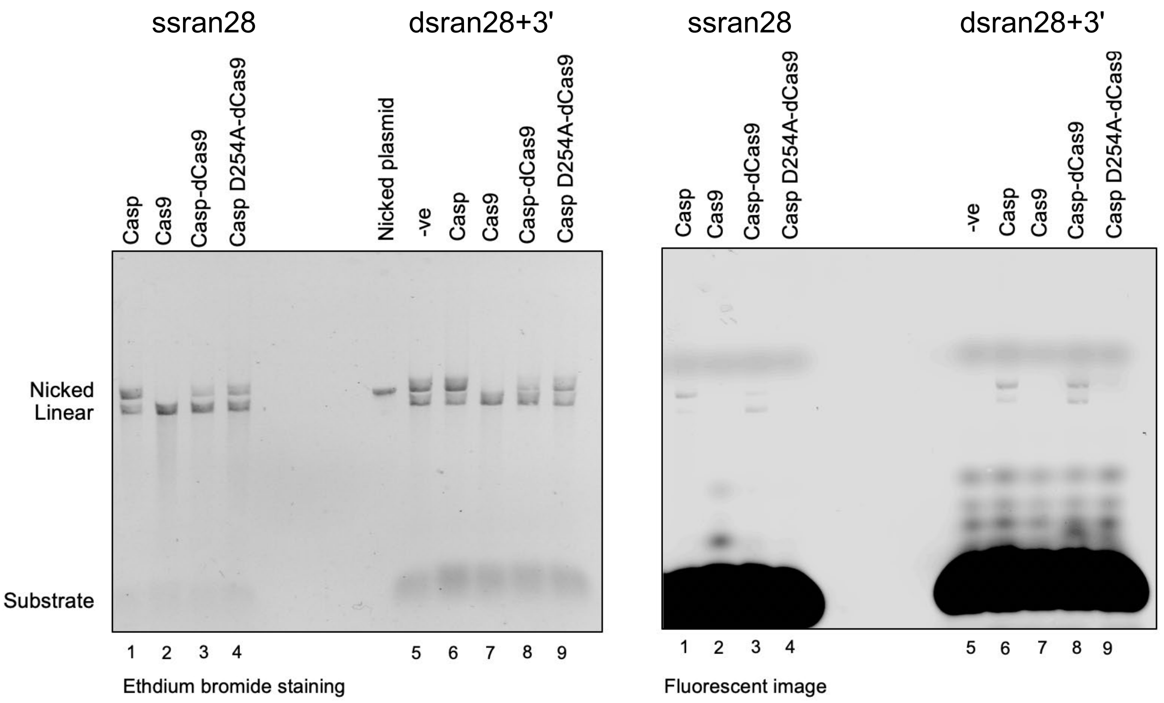

Figure S12. Upstream and target site sequences of sites A and B were aligned to the sequence logo reported in Figure 1F and *A. boonei* tRNA-Pro gene, where the casposon was inserted.

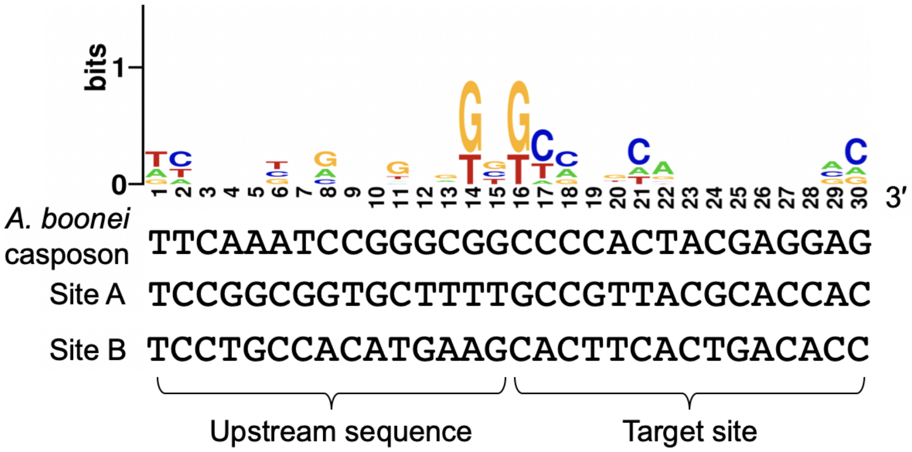

Figure S13. Uncropped image of the western blot from Figure 4B.

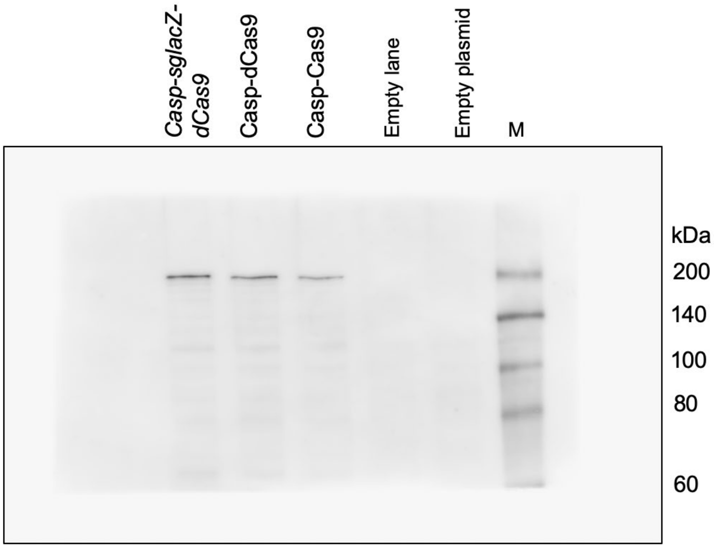

Figure S14. Viability spot test of *E. coli* cultures used in Miller assays. At cell culture OD_{600} of 0.6 immediately prior to harvesting cells for the Miller assay, 10μL of culture was diluted to 10^{-6}, 10^{-7}, 10^{-8} and 10μL of each spotted onto LB agar plates. (A) *E. coli* MG1655 K-12 Δ*lacZ* cells with inducible T7 RNA polymerase was transformed with pRC7 and different protein plasmids. (B) *E. coli* MG1655 K-12 cells with inducible T7 RNA polymerase was transformed with different protein plasmids.

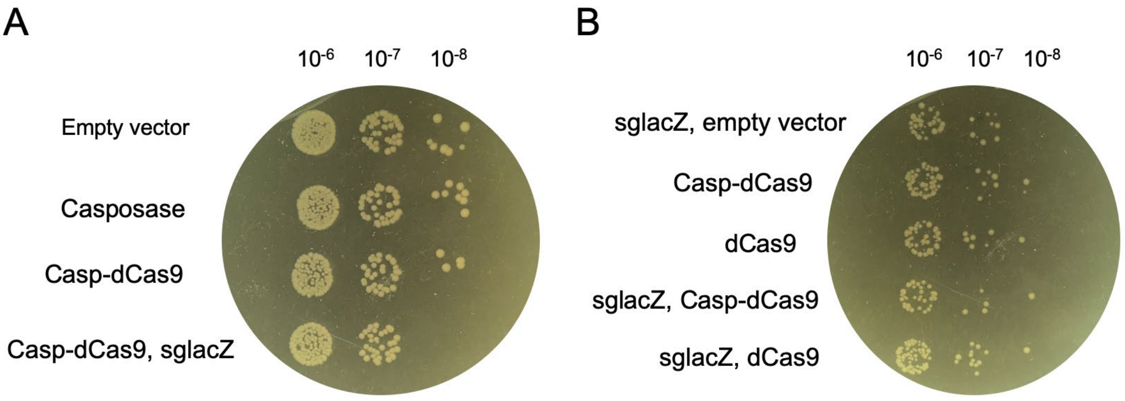

Figure S15. Casposase catalysed in vitro DNA integration in the presence of human cell-free extract. (A) Quantification of band intensity from dsran28+3' DNA integration catalysed by casposase in different reaction conditions. The band intensity was normalised to reactions using $MnCl_2$ buffer and without cell-free extract to obtain relative integration efficiency. Error bars represent standard error of the mean. N=3. One-way ANOVA with multiple comparison was performed to determine statistical significance. (B) A representative gel for quantification in (A). Casposase at 150 nM was incubated with 250 ng pACYC-Duet and 100 nM Cy5 labelled dsran28+3' in $MnCl_2$ containing integration buffer used in previous *in vitro* assays or in the same buffer with 5 mM $MgCl_2$ substituting for 5 mM $MnCl_2$. The reactions were also repeated in the presence of 18 µg of U2OS cell-free extract. Integration of dsran28+3' into pACYC-Duet was assessed using ethidium bromide to indicate supercoiled, linear and nicked plasmids (right), and fluorescence to detect Cy5 labelled dsran28+3' that was integrated in to the plasmid (left).

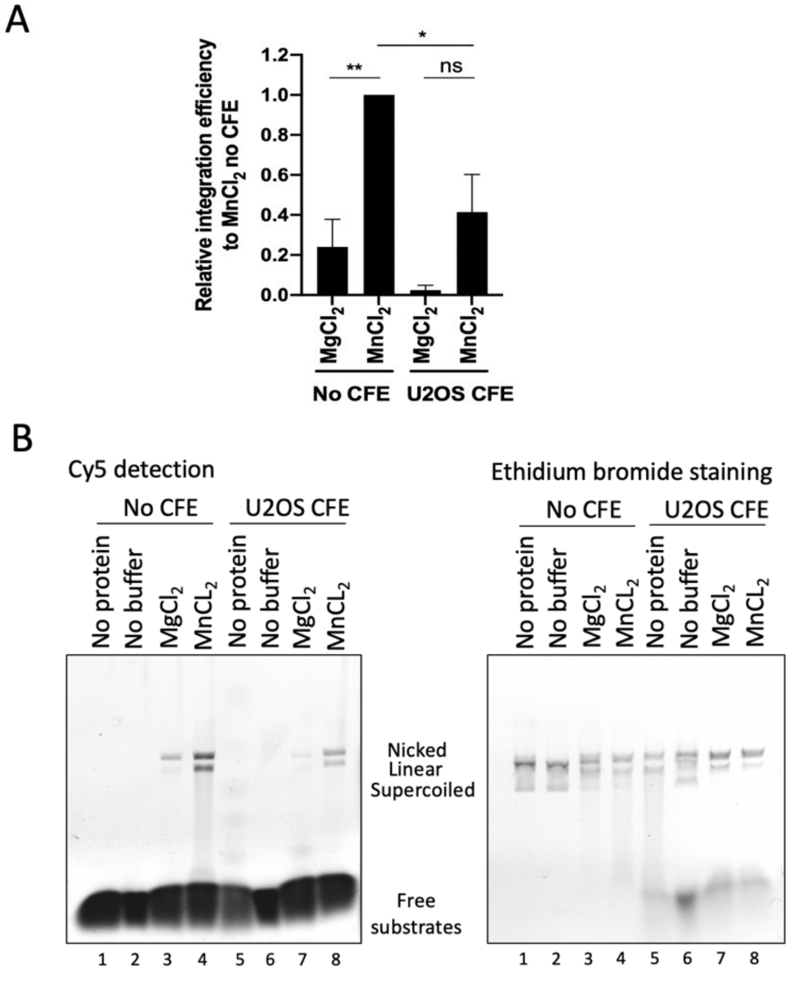

Supplement: Supplementary Figures S1-15- Tables S1-S4 [file BSR-2020-3595_supp.pdf]
